# The non-steroidal mineralocorticoid receptor antagonist finerenone and heart failure with preserved ejection fraction

**DOI:** 10.1186/s12933-023-01899-0

**Published:** 2023-06-29

**Authors:** Ulrich Kintscher, Frank Edelmann

**Affiliations:** 1 Institute of Pharmacology, Charite - Universitätsmedizin Berlin, corporate member of Freie Universität Berlin and Humboldt- Universität zu Berlin, Max Rubner Center for Cardiovascular Metabolic Renal Research, Hessische Str. 3-4, 10115 Berlin, Germany; 2 DZHK (German Centre for Cardiovascular Research), partner site Berlin, Berlin, Germany; 3 Deutsches Herzzentrum der Charité, Campus Virchow-Klinikum, 13353 Berlin, Germany; 4 Berlin Institute of Health, 13353 Berlin, Germany

**Keywords:** steroidal/ non-steroidal mineralocorticoid receptor antagonist, Heart failure with preserved ejection fraction, Finerenone, Cardiac fibrosis, Diastolic dysfunction

## Abstract

Finerenone is a novel non-steroidal mineralocorticoid receptor (MR) antagonist (MRA) with high binding affinity, high MR selectivity and a short plasma half-life. In two major endpoint-driven clinical trials in patients with chronic kidney disease and type 2 diabetes mellitus (FIDELIO-DKD and FIGARO-DKD), finerenone induced significant cardiorenal protective actions, and has been recently approved for treatment of these patients. Heart failure with preserved ejection fraction (HFpEF) is a devastating clinical syndrome with increasing prevalence and poor prognosis. Pharmacological therapy of HFpEF is very limited and new therapeutic options are urgently needed. Finerenone has been shown to improve multiple pathophysiological parameters of HFpEF in preclinical models. In consonance, pre-specified subgroup analyses of FIDELIO-DKD and FIGARO-DKD suggested a potential beneficial effect of finerenone in HFpEF. This review will discuss the pharmacodynamic and -kinetic profile of finerenone. We will provide a general overview over the complex pathophysiology of HFpEF and data from pre-clinical studies, focusing on how finerenone improves multiple components of this pathophysiology. Finally, we will discuss current and future clinical trials with finerenone in heart failure patients focusing on HFpEF.

## Introduction

The mineralocorticoid receptor (MR) belongs to the family of nuclear hormone receptors [1]. Pharmacological blockade of the MR by MR-antagonists (MRA) in renal epithelial cells results in a reduction of sodium and water reabsorption [2]. More importantly, inhibition of MR activity in cardiovascular cells including cardiomyocytes or fibroblasts, and in cells of the innate and adaptive immune system mediates potent anti-hypertrophic, anti-fibrotic and anti-inflammatory actions thereby improving cardiac function [3]. MRA, in particular steroidal MRA are routinely used to treat primary hyperaldosteronism, heart failure with reduced ejection fraction (HFrEF) and therapy-resistant hypertension [3]. Steroidal MRA have become one of the four first-line therapies for HFrEF, next to angiotensin converting enzyme inhibitors (ACE-I) or angiotensin receptor-neprilysin inhibitor (ARNI), beta-blockers and sodium-glucose co-transporter 2 (SGLT2) inhibitors [4]. Their role in HFpEF therapy is less clear and will be discussed in this review.

Recently, a new class of MRA has been developed, named non-steroidal MRA, among which finerenone is the one with the most advanced global clinical development program [5]. In two phase 3 clinical trials (FIDELIO-DKD and FIGARO-DKD), finerenone improved the cardiovascular and renal outcome of patients with chronic kidney disease (CKD) and type 2 diabetes mellitus (T2DM) significantly [6, 7]. These results led to the approval of finerenone by the FDA and the EMA in 2021/22 for the treatment of patients with CKD and T2DM to reduce the risk of kidney function decline, kidney failure, CV death, nonfatal heart attacks and hospitalization for heart failure. Compared to steroidal MRA, finerenone exerts a different binding mode to the MR-ligand binding domain, as a bulky antagonist, thereby leading to distinct MR-target gene regulation which translates into distinct cardiorenal effects and clinical actions [8–11]. In preclinical and clinical studies, finerenone has been shown to induce cardioprotective actions, to decrease levels of BNP and NT-proBNP and to reduce the risk of incident heart failure in patients with CKD and T2DM [5, 12]. This review will discuss preclinical and clinical data on finerenone in heart failure focusing on its future role in HFpEF therapy.

HFpEF is a clinical syndrome accounting for over 50% of the estimated 64.3 million heart failure patients worldwide [13]. The 5-year survival rate of HFpEF patients is 69.8% and is not significantly different from patients with HFrEF [14]. The underlying pathophysiology is multifactorial and mainly driven by non-cardiac conditions and comorbidities such as hypertension, T2DM, obesity, and CKD, among which hypertension is one of the most prevalent [15–18]. So far therapeutic options to reduce morbidity and mortality in HFpEF are very limited. Recently, SGLT2-inhibitors showed significant benefit on heart failure hospitalization rates and are now recommended in patients with HFpEF [19]. New effective therapeutic options are imperative to alleviate the suffering of these patients. Based on their mechanism of action, on preclinical data and post-hoc analysis of clinical trials, MRA represent a promising alternative for future HFpEF therapy.

In summary, this review will discuss the pharmacology of the novel non-steroidal MRA, finerenone, and its potential use for the treatment of patients with HFpEF.

## Mineralocorticoid receptor antagonists (MRA)

### Steroidal MRA

Nowadays, two steroidal MRA are in routine clinical use, spironolactone and eplerenone [3, 20, 21]. Despite a similar binding mode to the MR, as so-called ‘passive’ antagonists, they reveal important differences in their pharmacodynamic and pharmacokinetic properties (Table 1) [3, 5]. Spironolactone is a high-affinity ligand with lower MR selectivity, whereas eplerenone has a lower MR-affinity with higher selectivity (MR IC_50_: spironolactone: 24nM and eplerenone: 990nM) (Table 1) [3]. Accordingly, eplerenone develops fewer sex steroid hormone receptor-mediated side effects such as gynecomastia due to unspecific binding when compared to spironolactone (Table 1) [3]. Eplerenone has a shorter plasma half-life than spironolactone, which is metabolized to active compounds, canrenone and 7alpha-thiomethylspironolactone (plasma half-life: spironolactone > 20 h and eplerenone 4-6 h) (Table 1) [22]. Steroidal MRA are now first-line therapy in patients with HFrEF [4]. This recommendation is based on the positive results in previous randomized clinical trials including RALES, EPHESUS and EMPHASIS. Spironolactone was tested in 1.663 patients with HFrEF, NYHA stage III-IV, in the Randomized Aldactone Evaluation Study (RALES) [5, 23]. Spironolactone significantly reduced mortality and markedly lowered hospitalization rate for heart failure in RALES [5, 23]. The Eplerenone Post-Acute Myocardial Infarction Heart Failure Efficacy and Survival Study (EPHESUS) demonstrated a significant reduction of total-/ cardiovascular mortality and hospitalization rate by eplerenone in 6.642 patients with myocardial infarction, LV-EF ≤ 40% and symptomatic heart failure [5, 24]. And in 2.737 patients with LV-EF ≤ 30% and NYHA stage II, eplerenone treatment induced a significant decline of all-cause and CV death, and hospitalization rate in the Eplerenone in Mild Patients Hospitalization and Survival Study in Heart Failure (EMPHASIS) [5, 25]. Regarding safety of steroidal MRA, one of the main side effects and thus often therapy-limiting, is hyperkalemia. Specifically, when combined with other blockers of the renin-angiotensin-aldosterone system such as ACE-I, angiotensin receptor blockers or ARNIs, and in the presence of CKD the risk for hyperkalemia increases severalfold with steroidal MRA [26].

**Table 1:** [5] Key pharmacodynamic- and kinetic parameters of steroidal MRA and finerenone. MR: mineralocorticoid receptor; MRA: MR-antagonist; CNS: central nervous system; BP: blood pressure

	Steroidal MRA		Non-steroidal MRA
	Spironolactone	Eplerenone	Finerenone
Structural properties	Flat (steroidal)	Flat (steroidal)	Bulky (non-steroidal)
MR-Affinity	+++	+	+++
MR-Selectivity	+	++	+++
Sex-hormone-related side effects	++	(+)	-
CNS penetration	+	+	-
Plasma half-life	> 20 h	4-6 h	2-3 h
Effect on BP	+++	++	+

In summary, there is compelling evidence for the use of steroidal MRA in HFrEF to improve morbidity and mortality in these patients. However, this significant efficacy is associated with an increased risk of developing hyperkalemia, which requires close monitoring. In contrast, the evidence in HFpEF for MRA use is currently much less convincing and is discussed below.

### Non-steroidal MRA

To further improve the benefit-risk profile of MR-based therapy, new compounds without a steroidal molecule backbone have been recently developed and named non-steroidal MRA [27]. Their discovery and development have been accurately reviewed elsewhere [3, 5, 22, 27–30]. Multiple compounds such as finerenone, esaxerenone, AZD9977, apararenone, and KBP-5074, are currently in clinical development for different indications including arterial hypertension, uncontrolled hypertension and CKD, T2DM and CKD, HFrEF and HFpEF [3, 5, 22, 27–30].

In differentiation from steroidal MRA, most of these compounds appear to have a different binding mode to the MR-ligand binding domain (LBD), thereby inducing distinct pharmacodynamic actions [5]. This new class of MRA exhibit high selectivity for the MR and does not mediate sex steroid-related side effects [5]. They differ in their MR-binding affinity, with KBP-5074, esaxerenone and finerenone exhibiting a high MR-binding affinity, and AZD9977 and apararenone a lower binding affinity [9, 31–34]. In addition, the plasma half-life of the compounds differs significantly: finerenone 2-3 h, AZD9977 4-9 h, esaxerenone 18 h, KBP-5074 60 h, and apararenone 375-285 h [5, 32, 34–37].

With respect to their clinical efficacy esaxeronone, approved for the treatment of arterial hypertension in Japan, and KBP-5074 induce significant antihypertensive actions ranging between − 11.5mmHg systolic blood pressure (SBP) lowering for low doses and − 16.5mmHg for high doses [35, 38]. Significant renoprotective actions have been described for esaxerenone and finerenone [6, 7, 39]. The most advanced clinical development program has been published for finerenone demonstrating a clear cardiorenal benefit in patients with CKD and T2DM (see details below) [6, 7]. Regarding heart failure therapy, in addition to the studies with finerenone which will be described in detail, only one study with AZD9977 is currently underway. In this study, patients with heart failure with an ejection fraction < 60% and CKD (eGFR ≥ 20 to ≤ 60ml/min/1.73m^2^) will be treated with different doses of AZD9977 in combination with the SGLT2-inhibitor dapaglifozin (10 mg) or dapaglifozin (10 mg) alone (NCT04595370). The estimated enrollment will be 500 patients and the estimated study completion date is in December 2023 (www.clinicaltrials.gov). Focusing on safety issues with novel non-steroidal MRA, most of the compounds induce hyperkalemia when compared to placebo, however, when compared to steroidal MRA this occurs to a lesser extent [5].

### Finerenone

Finerenone is a novel non-steroidal MRA with high binding affinity (IC50: 18nM) and high MR selectivity over sex steroid hormone receptors, thereby lacking anti-androgenic and progestational side effects (Table 1) [9, 22]. Finerenone has s short plasma half-life between 2 and 3 h (Table 1) [36]. Its specific binding mode to the MR-LBD as a so called “bulky” antagonist induces a distinct MR-LBD conformational change with protrusion of Helix 12, an unstable receptor-ligand complex and prevention of transcriptional coregulator recruitment (Table 1) [8, 9, 11]. This ligand-specific coregulator binding to the MR results in ligand-specific gene regulation explaining, at least in part, some of the clinical actions observed with finerenone [8, 9, 11].

After completion of a successful phase I/II clinical development program, two phase III clinical trials with finerenone were conducted in patients with T2DM and CKD. In FIDELIO-DKD, 5.734 patients with CKD (UACR 30 to < 300 mg/g and eGFR ≥ 25 to < 60mL/min/1.73m^2^ or UACR ≥ 300 mg/g and eGFR ≥ 25 to < 75 mL/min/1.73m^2^) and T2DM were randomized to placebo or finerenone (10 or 20 mg/d) [5, 6]. All patients were treated with RAS-blockade (ACE-inhibitors or ARBs) at the maximum tolerable dose and over 95% had hypertension [5, 6]. After a median follow-up of 2.6 years, finerenone significantly lowered the primary composite outcome (time to onset of kidney failure, sustained decrease of eGFR ≥ 40% from baseline or renal death) compared to placebo (hazard ratio, 0.82; 95% confidence interval [CI], 0.73 to 0.93; P = 0.001) [5, 6]. Additionally, the secondary outcome event (time to CV death, nonfatal MI or stroke, or hospitalization for heart failure) occurred significantly less in the finerenone group (hazard ratio, 0.86; 95% CI, 0.75 to 0.99; P = 0.03) [5, 6]. The incidence of hyperkalemia-related discontinuation of the trial regimen was higher with finerenone (2.3%) compared to placebo (0.9%), but lower than in other trials with dual RAAS-blockade [5, 6].

The second phase III trial, FIGARO-DKD was conducted in a similar patient population as FIDELIO-DKD and focusses with its primary CV-composite endpoint (time to CV death, nonfatal MI or stroke, or hospitalization for heart failure) on CV morbidity and mortality [5, 7]. 7.437 randomized patients had earlier stage CKD compared to FIDELIO-DKD, defined by UACR 30 to < 300 mg/g and eGFR 25 to ≤ 90mL/min/1.73m^2^ or UACR ≥ 300 mg/g and eGFR ≥ 60mL/min/1.73m^2^) and T2DM [5, 7]. The renal key secondary endpoint replicated the primary composite of FIDELIO-DKD [5, 7]. All patients were treated with optimal RAS-blockade and over 95% had hypertension [5, 7]. Patients are randomized to placebo or finerenone (10-20 mg/d) [5, 7]. After a median follow-up of 3.4 years finerenone significantly reduced the primary CV-composite endpoint compared to placebo (hazard ratio, 0.87; 95% confidence interval [CI], 0.76 to 0.98; P = 0.03) [5, 7]. The secondary composite outcome (time to onset of kidney failure, sustained decrease of eGFR ≥ 40% from baseline or renal death) occurred in 350 patients (9.5%) in the finerenone group and in 395 (10.8%) in the placebo group (hazard ratio, 0.87, 95% CI, 0.76–1.01) [7].

Drug-drug interactions with finerenone have been investigated in healthy male volunteers [40]. Finerenone is predominantly metabolized by CYP3A4 in the gut wall and liver [40]. Accordingly, concomitant use of finerenone with strong CYP3A4 inhibitors such as ketoconazole, ritonavir, nelfinavir, cobicistat, telithromycin or nefazodone is contraindicated since a marked increase in finerenone exposure is expected [41]. Grapefruit or grapefruit juice (both CYP3A4 inhibitors) should not be consumed during finerenone [41]. Furthermore, finerenone should not be used concomitantly with strong or moderate CYP3A4 inducers (e.g. rifampicin, carbamazepine, phenytoin, phenobarbital, St. John´s Wort) which are expected to markedly decrease finerenone plasma concentration [41]. Other drugs should be used with precautions during finerenone treatment including erythromycin, verapamil and fluvoxamine since increased plasma concentrations have been reported increasing the risk of hyperkalemia [41]. Because of an increased risk for hyperkalemia, finerenone should not be co-administered with potassium-sparing diuretics (e.g. amiloride or triamterene) or with other MRAs, and monitoring of potassium is required with the concomitant use of potassium supplements and trimethoprim, or trimethoprim/ sulfamethoxazole [41]. The risk of hypotension increases with concomitant use of other antihypertensive drugs [41]. The combination therapy of finerenone with sodium glucose co-transporter 2 (SGLT2) inhibitors has been recently discussed [42]. It seems that this combination mediates additional beneficial actions in terms of further reducing cardiorenal risk and attenuating side effects, especially hyperkalemia [42]. Prospective randomized clinical trials are currently being conducted to demonstrate the benefit of such a combination therapy [43].

Recent publications confirmed the protective actions of finerenone in cardiorenal disease. A large network meta-analysis showed that in patients with T2DM and CKD, finerenone led to a risk reduction in MACE, renal outcomes and hospitalization for heart failure [44]. Certain patient subgroups including non-albuminuric CKD patients seem to be usually underrepresented in the finerenone RCTs [45]. In this regard, the FIDELITY prespecified pooled analysis of the combined data from FIDELIO-DKD and FIGARO-DKD revealed that finerenone reduced the risk of clinically important cardiovascular and kidney outcomes across the spectrum of CKD in patients with T2DM [46]. Furthermore, compared with placebo, finerenone improved heart failure-related outcomes in patients with CKD and T2DM, with consistent benefit across eGFR and/ or UACR [47]. In summary, these data convincingly demonstrate that the non-steroidal MRA finerenone induces cardiorenal protection in patients with CKD and T2DM which led to its approval by the FDA and EMA for the treatment of adults with CKD and T2DM to reduce the risk of kidney function decline, kidney failure, CV-death, non-fatal heart attacks, and hospitalization for heart failure.

### Heart failure with preserved ejection fraction (HFpEF)

**Fig. 1:**
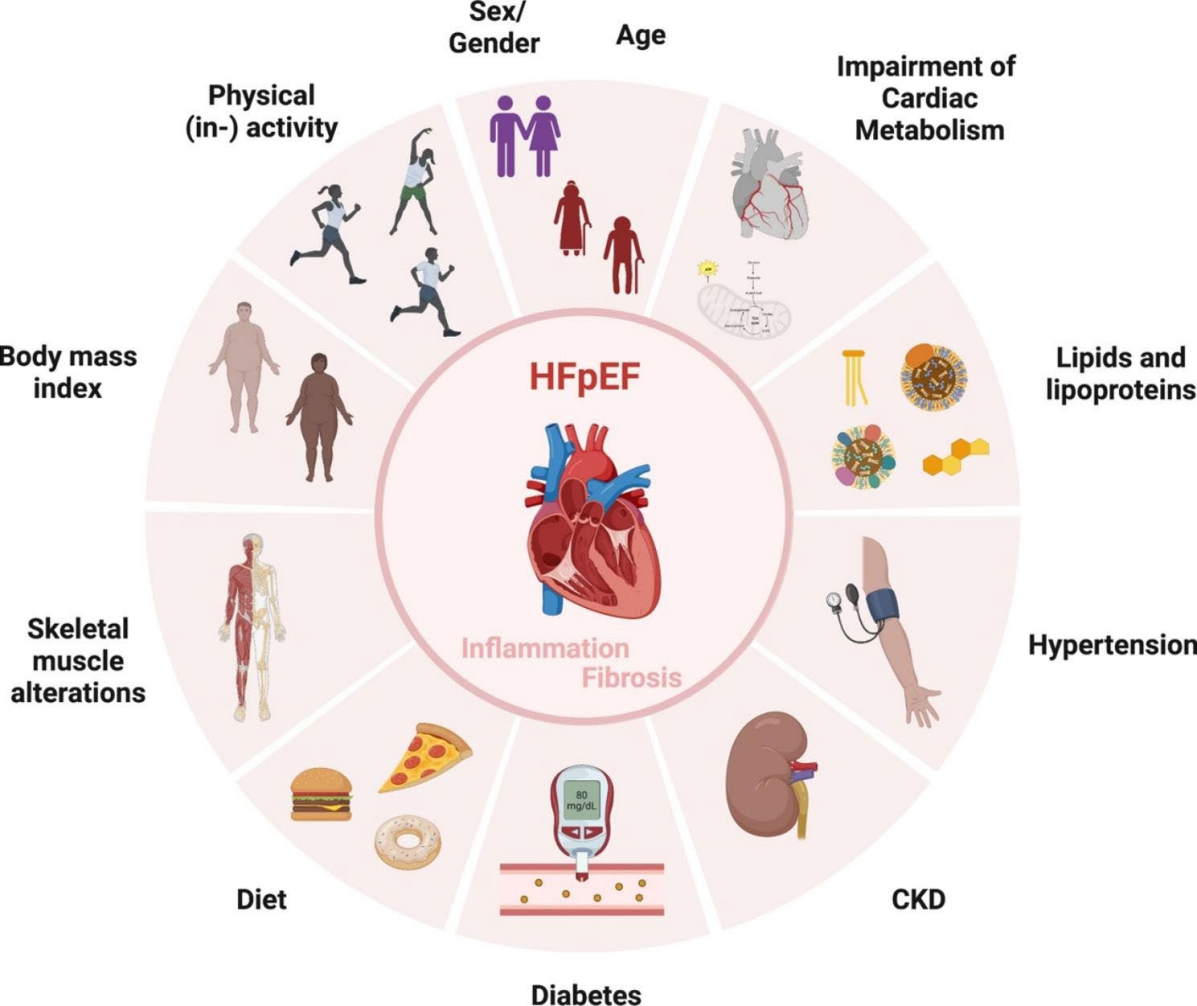
Pathophysiological drivers and comorbidities in heart failure with preserved ejection fraction (HFpEF). CKD: chronic kidney disease. [adapted from “Risk Factors of Dementia” template, by BioRender.com (2022). Retrieved from https://app.biorender.com/biorender-templates]

HFpEF is a clinical syndrome that now accounts for almost half of all heart failure cases with an increasing prevalence [13]. The pathophysiology is multifactorial and is driven by known comorbidities such as hypertension, T2DM, obesity, CKD, and other pathological non-cardiac/ cardiac factors among which hypertension is the most prevalent (Fig. 1) [48, 49]. To improve diagnostic characterization of HFpEF patients, so called phenogroups have been defined based on large scale clinical/ biochemical parameters analyzed with novel statistical learning methods [17, 50, 51]. Multiple HFpEF phenotypes have been specified including an *older vascular aging* phenotype, a *metabolic obese* phenotype and relatively *younger natriuretic peptide (NP) deficient* phenotype [50]. These overlapping HFpEF phenotypes occur with varying frequencies in phenomapping studies such as the *older vascular aging* phenotype in 30–50%, the *metabolic obese* in 25–30% and the *younger NP deficient* phenotype in 15–35% of HFpEF patients [50]. To establish such clinical phenotyping approaches in clinical routine, the phenogroups need to be further validated in additional clinical studies and machine-learning methods must be integrated into everyday clinical practice [50]. However, it seems that new pathophysiological concepts are more and more aligned with the aforementioned phenogroups. Thus, the *older vascular aging* phenotype appears to be driven by noncardiac factors, such as vascular stiffness or CKD-associated renal factors in combination with cardiac factors precipitating the development of cardiac hypertrophy, left atrial myopathy, and right ventricular dysfunction[50]. On the other hand, the *metabolic obese* phenotype is provoked by noncardiac factors, such as systemic fat accumulation during obesity and/or impaired insulin/ glucose metabolism, as well as by direct alterations of cardiac metabolism [50]. Disturbances of cardiac metabolism in HFpEF include impaired mitochondrial ATP production associated with altered mitochondrial morphology including reduced mitochondrial area, mitochondrial fragmentation and cristae destruction, mitochondrial swelling and vacuolar degeneration, and impaired mitochondrial function (reduced complex I and II activity) [48, 50]. Furthermore, cardiac metabolic flexibility is lost in HFpEF which goes along with increased rates of anaerobic glycolysis, a shift towards the hexosamine pathway, and increased fatty uptake paralleled by impaired fatty acid oxidation resulting in enhanced cardiac triacylglycerol, diacylglycerol, and ceramide storage [48]. Finally, the *younger NP deficient* phenotype is characterized by reduced NP production and increased NP clearance and might be driven by perturbations in the cell – matrix compartment since increased levels of syndecan-4 and matrix metalloproteinase (MMP)-9 have been detected [50]. With increasing understanding of the aforementioned phenogroups and their underlying pathophysiology, new personalized therapeutic strategies could then be developed in the future.

When discussing the pathophysiology of HFpEF, the importance of arterial hypertension should be emphasized. The prevalence of hypertension among patients with HFpEF ranges between 28% and 90% [52–55]. In TOPCAT, PARAGON-HF, and EMPEROR-Preserved even more than 90%, and in FIDELIO-DKD and FIGARO-DKD more the 95% of the included patients suffered from arterial hypertension underscoring the importance of high blood pressure in the pathophysiology of HFpEF [6, 7, 15, 16, 18]. The high relevance of an effective antihypertensive treatment in patients with HFpEF and hypertension has been recently reiterated [53]. However, despite the high prevalence of hypertension in HFpEF, this clinical syndrome differs from hypertensive heart disease by multiple pathophysiological mediators evidenced by the described clinical identification of variable HFpEF phenotypes that go beyond hypertension-mediated pathology [48, 49, 51].

A central pathophysiological pathway that probably overlaps in all phenogroups is the inflammatory – fibrotic pathway [56]. A subclinical chronic systemic inflammation has been detected in HFpEF, and likely stimulates the invasion of the heart with immunocompetent cells [56]. This process results in the decline of endothelial NO-production and an induction of endothelial production of reactive oxygen species [56]. Low NO and high ROS causes alterations of titin leading to cardiomyocyte stiffness and diastolic dysfunction [56]. In addition, infiltrating monocyte/ macrophages stimulate the collagen production by cardiac fibroblasts[56]. The central importance of these proinflammatory-profibrotic events, makes these processes highly attractive as therapeutic targets. Interestingly, (pre-) clinical evidence exists that MRA and specifically finerenone exert potent anti-inflammatory and anti-fibrotic activity, and these are highly suggestive as pharmacological interventions in HFpEF [3].

Current drug therapy for HFpEF is very limited. SGLT-2 inhibitors are recommended as first-line therapy since they improve prognosis [4]. Two randomized clinical trials of 5.988 (EMPEROR-Preserved) and 6.263 (DELIVER) patients with HFpEF showed that the SGLT-2 inhibitors empaglifozin and dapaglifozin significantly reduce the primary composite endpoint of hospitalization for heart failure and cardiovascular death [15, 49, 57]. It should be noted that the benefit in both trials was due to a reduction in heart failure hospitalization rate, without a significant reduction in cardiovascular mortality [15, 49, 57]. In addition to this prognosis-improving therapy, symptom-oriented therapy is currently the basis of pharmacological HFpEF therapy, including diuretic therapy in the presence of congestion or blood pressure lowering therapy for hypertension [4].

In summary, new therapeutic approaches to improve the prognosis of HFpEF are imperative and based on our current pathophysiological knowledge and post-hoc analysis of major event-driven clinical trials, we will discuss the potential benefit of MR-blocking drugs in this context.

### MRA therapy in HFpEF

Steroidal MRA are first-line therapy in HFrEF together with ACE-I or ARNI, beta-blocker and SGLT2-inhibitors [4]. Their role in HFpEF therapy is less clear.

Spironolactone was investigated in 3.445 HFpEF patients (EF ≥ 45%) in the TOPCAT trial [16]. After a mean follow-up of 3.3 years, the primary composite outcome (cardiovascular death, aborted cardiac arrest, hospitalization for heart failure) occurred in 18.6% patients in the spironolactone group and in 20.4% in the placebo group not reaching statistical significance (hazard ratio, 0.89; 95CI, 0.77 to 1.04; P = 0.14) [16]. Hospitalization for heat failure was significantly lower in the spironolactone group compared to placebo (206 patients [12.0%] vs. 245 patients [14.2%]; hazard ratio, 0.83; 95%CI, 0.69 to 0.99; P = 0.04) [16]. However, a post-hoc analysis of TOPCAT revealed that substantial regional differences in study outcome occurred between patients recruited in Russia/ Georgia (n = 1.767) and those recruited in the Americas (n = 1.678). In contrast to Russia/ Georgia, where all clinical event rates were markedly lower, in the Americas Spironolactone significantly reduced the primary outcome [58]. Furthermore, a sub-study in 366 patients from the TOPCAT trial measured serum concentrations of the spironolactone metabolite canrenone and demonstrated that among the participants who were assigned to receive spironolactone, canrenone concentrations were undetectable in a higher percentage of participants from Russia than from the United States and Canada [59]. These data indicate important regional variations in drug adherence during the trial, which may, at least in part explain the negative results in the overall trial population [16, 59]. Along this line, it has been suggested by Pfeffer and Braunwald that based on these findings in TOPCAT in North and South America and in the absence of more definitive data, it now appears reasonable to treat patients with HFpEF resembling those enrolled in North and South America with spironolactone to improve outcomes [60]. Furthermore, based on post-hoc analysis from TOPCAT, the potential efficacy of spironolactone in HFpEF patients seems to be greater at the lower end of the LV-EF spectrum [61, 62].

In addition to TOPCAT, spironolactone has been tested in the Aldo-DHF (Aldosterone Receptor Blockade in Diastolic Heart Failure) trial which included 422 ambulatory patients with HFpEF (EF ≥ 50%) and diastolic dysfunction randomized to spironolactone or placebo [63]. After a 12-month follow-up, spironolactone significantly improved diastolic dysfunction (E/e’) and reduced LV-mass index (LVMI) but did not affect maximal exercise capacity, patient’s symptoms, or quality of life [63]. In a recent individual patient-data meta-analysis of Aldo-DHF, TOPCAT and the HOMAGE (Heart Omics in AGEing) trial with 984 patients, it was demonstrated that spironolactone significantly reduced LAVI (left atrial volume index), LVMI, interventricular septal thickness, and E/e’ corroborating the beneficial actions of spironolactone on cardiac structure in patients with HFpEF [64].

In light of these results, the final efficacy evaluation of steroidal MRA in HFpEF appears to be ongoing and requires additional studies. Currently, two major ongoing studies are investigating the role of spironolactone in HFpEF (Table 2). The first study is a registry-based prospective randomized clinical trial (RRCT) called SPIRRIT-HFpEF (Spironolactone Initiation Registry Randomized Interventional Trial in Heart Failure with Preserved Ejection Fraction, NCT02901184) being conducted with the Swedish Heart Failure Registry and in the US with an estimated enrollment of 2.000 patients [27, 65]. The primary outcome is time to death from any cause and secondary measures are cardiovascular death or time to heart failure hospitalization [27, 65]. The second study, SPIRIT-HF (Spironolactone In The Treatment of Heart Failure, NCT04727073) is a study funded by the German Centre for Cardiovascular Research (DZHK)/ German Federal Ministry of Education & Research and investigates the effect of spironolactone on the composite endpoint of recurrent hospitalization for heart failure and CV death in 1.300 symptomatic heart failure patients (NYHA II-IV) with mid-range (LVEF ≥ 40–49%) or preserved (LVEF ≥ 50%) ejection fraction in Austria, France, Germany, and the Netherlands [27, 66].

**Table 2:** Ongoing clinical trials with spironolactone or finerenone in HFpEF.

Drug	Study Name NCT#	Condition / Disease	Estimated/ Actual Enrollment	Primary endpoint	Study start / completion
Spironolactone	SPIRRIT-HFPEF NCT02901184	HFmrEF (EF ≥ 40–49%) + HFpEF (EF ≥ 50%)	2.000	Time to death from any cause	November 2017/ December 2026
Spironolactone	SPIRIT-HF NCT04727073	HFmrEF (EF ≥ 40–49%) + HFpEF (EF ≥ 50%)	1.300	Cumulative number of primary composite events of CV death and total HF hospitalization	November 2018/ December 2024
Finerenone	FINEARTS-HF NCT04435626	HFmrEF (EF ≥ 40–49%) + HFpEF (EF ≥ 50%)	6.016	Number of cardiovascular death and heart failure events	September 2020/ August 2024

Abbreviations: CV: Cardiovascular, EF: Ejection fraction, HF: Heart failure, HFmrEF: Heart failure with midrange ejection fraction, HFpEF: Heart failure with preserved ejection fraction

These aforementioned results are also in line with further post-hoc analyses of the TOPCAT trial investigating whether MRA therapy may be more effective in specific HFpEF-phenogroups [50, 67–69]. Data from these studies implicate that spironolactone may have more favorable actions in the *metabolic obese* and the *younger NP deficient* phenotype [50, 67–69]. These results are highly significant for the development of future therapeutic strategies for HFpEF, as they point towards a personalized approach combining deep diagnostic phenomapping and pharmacological decision making, e.g. personalized MRA therapy.

One of the major limitations of the use of steroidal MRA in heart failure therapy is the development of hyperkalemia, specifically in patients with existing RAS inhibiting medication or in the presence of CKD. Thus, a 3.3-fold increased risk for the development of hyperkalemia was detected for spironolactone in the TOPCAT (America) trial [70]. An increased risk for hyperkalemia with MRA was recently confirmed in an individual patient data meta-analysis including 12.700 patients from RALES, EMPHASIS-HF, TOPCAT (Americas), and EPHESUS [71]. The limited benefit-risk profile of steroidal MRA in heart failure therapy led, among other reasons, to the development of non-steroidal MRA, such as finerenone, which so far appear to have a lower risk of hyperkalemia and thus may represent an alternative in heart failure/ HFpEF therapy.

## Finerenone and HFpEF

### Preclinical data

Finerenone is a novel non-steroidal, highly selective MRA with high binding affinity. It has been tested in various rodent models related to HFpEF. To draw conclusions from results of pre-clinical studies in HFpEF for clinical translation, strict criteria must be applied to the phenotyping of these HFpEF rodent models. Recently, Withaar and colleagues analyzed various rodent HFpEF models for their relevance to clinical HFpEF symptoms, clinical signs, and diagnostic criteria by applying two established clinical algorithms, the HFA-PEFF and H_2_FPEF score, to these models [72]. They found that most of the pre-clinical HFpEF models do not meet the HFpEF clinical criteria suggesting that there is currently no ideal rodent HFpEF model for clinical translation [72]. However, rodent models in which one or more of the individual components of HFpEF occur are available and have been tested with finerenone.

As described in detail above, inflammatory and fibrotic processes play a central pathophysiological role in the development of HFpEF. For Finerenone, significant anti-inflammatory and antifibrotic effects have been demonstrated in multiple pre-clinical studies. For instance, finereone reduced the cardiac accumulation of macrophages (CD68-positive cells) in a model of cardiac dysfunction induced by short-term isoproterenol application [11]. Potent anti-fibrotic actions of finerenone in the heart were documented in the deoxycorticosterone acetate (DOCA) salt - uninephrectomy rat and mouse model, the short-term isoproterenol model, and in the Zucker fa/fa rat model [11, 73–76]. Anti-fibrotic actions of MRAs have also been recently documented in clinical studies conducted with spironolactone demonstrating a significant reduction of serum concentrations of procollagen type 1 carboxy-terminal propetide, which reflects the synthesis of type 1 collagen and correlates with histologically proven cardiac fibrosis [77].

Cardiac hypertrophy is a key criterion for HFpEF in the HFA-PEFF score [78]. Finerenone has been shown to reduce cardiac hypertrophy in different rodent models including the pressure-induced transverse aortic constriction mouse model, the DOCA salt - uninephrectomy rat and mouse model, the short-term isoproterenol model, and in the Zucker fa/fa rat model [11, 73, 74, 76, 79]. The second key diagnostic parameter for HFpEF, which is part of the HFA-PEFF and the H_2_FpEF score, is the presence of diastolic dysfunction [78, 80]. Using invasive hemodynamic measurements, finerenone reduced LV-end diastolic pressure and/ or LV-end-diastolic pressure-volume relationship in different mouse and rat models indicating an improvement of LV diastolic dysfunction [74, 75, 81]. Furthermore, early signs of LV systolic dysfunction occur in HFpEF mainly caused by altered systolic function of subendocardial LV myofibers and detected in speckle tracking echocardiography by analysis of the global longitudinal strain (GLS) [78, 82]. Finerenone has been shown to significantly improve GLS in different mouse models [11, 76]. The action of finerenone on blood pressure has been investigated in some preclinical models of cardiac dysfunction/ heart failure demonstrating an overall antihypertensive effect [11, 73, 76, 81]. However, in all clinical studies the antihypertensive effects of finerenone were classified as modest compared to placebo and to a lesser extent when compared to spironolactone [5].

**Fig. 2:**
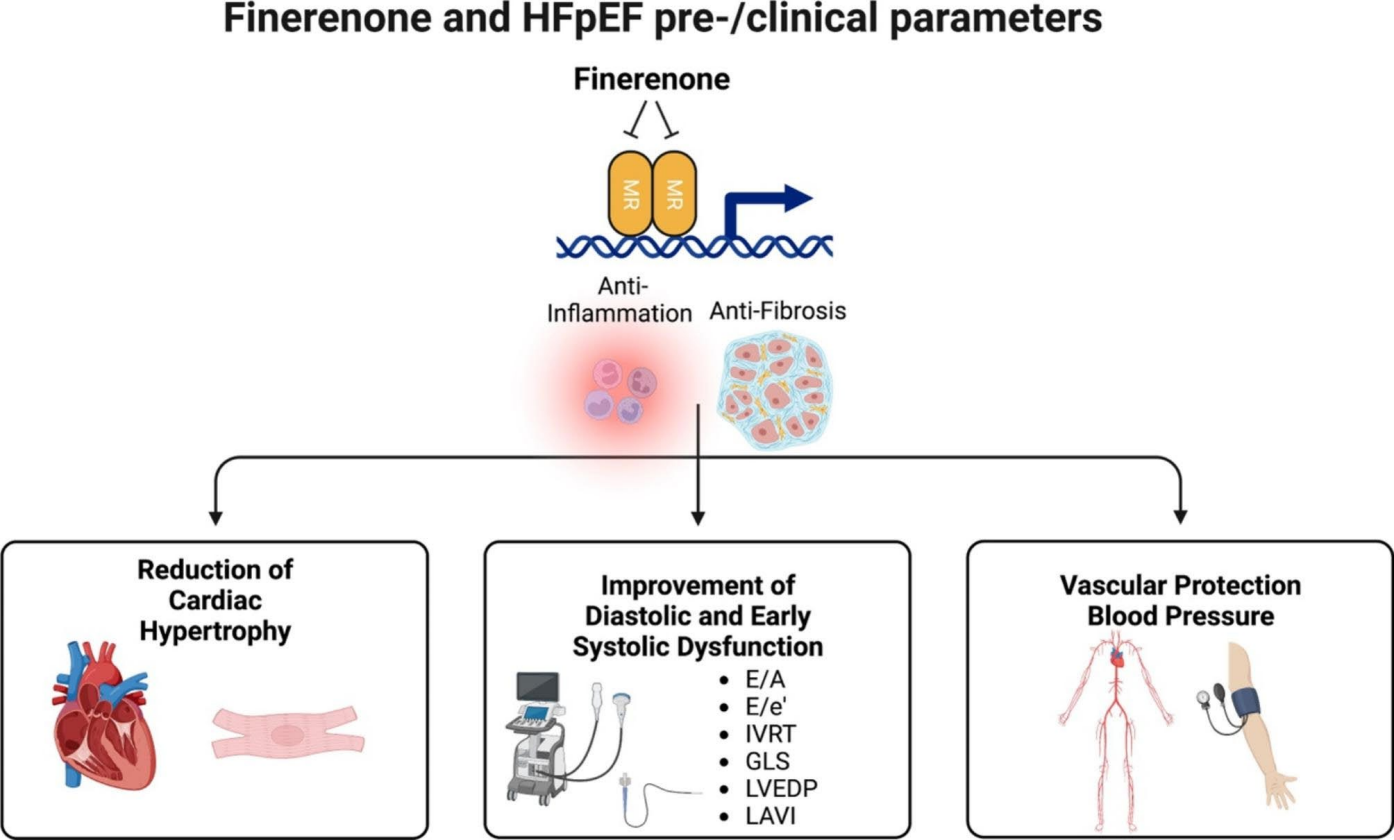
Cardioprotective actions of Finerenone in HFpEF. MR: mineralocorticoid receptor; E: early diastolic flow velocity; e’: velocity of early diastolic mitral annular motion; IVRT: isovolumic relaxation time; GLS: global longitudinal strain; LVEDP: left ventricular end-diastolic pressure. [adapted from “Mast Cell Key Features” template, by BioRender.com (2022). Retrieved from https://app.biorender.com/biorender-templates]

In summary, although finerenone has been tested in pathophysiologically/ phenotypically diverse cardiac injury models, a consistent anti-inflammatory/ anti-fibrotic effect was detected (Fig. 2). With respect to important clinical HFpEF criteria from current diagnostic scoring algorithms (HFA-PEFF and H_2_FPEF), finerenone exhibited beneficial actions on several of these parameters in preclinical studies including on cardiac hypertrophy, on diastolic dysfunction, on early alterations of systolic LV-function and on blood pressure regulation (Fig. 2). Considering these preclinical results together, finerenone appears to be a promising pharmacological alternative in HFpEF therapy, which needs to be proven in future clinical trials.

### Clinical data

One of the first signs of beneficial actions of finerenone on cardiac function in heart failure was published in the phase II safety/ tolerability study (ARTS) [83]. In one part of ARTS, 392 patients with HFrEF and moderate CKD were randomized to different doses of finerenone (BAY-948,862), open-label spironolactone or placebo [83]. Despite no significant overall treatment effect on BNP and NT-proBNP, the decreases in patients receiving finerenone were comparable with those recorded in the spironolactone group [83]. In addition, finerenone was associated with lower incidences of hyperkalemia than spironolactone [83]. In ARTS-HF, patients with HFrEF and T2DM and/or CKD were randomized to different doses of finerenone or eplerenone [5, 84]. All finerenone dose groups exhibited a similar proportion of patients with > 30% decline in NT-proBNP compared to the eplerenone group and the pre-specified exploratory composite CV clinical endpoint including all-cause death, CV-hospitalizations, or emergency presentation for worsening heart failure occurred numerically less frequently with finerenone compared to eplerenone [5, 84].

Two recent prespecified subgroup analyses of the FIDELIO-DKD and FIGARO-DKD studies investigated the efficacy of finerenone in patients with CKD and T2DM with and without heart failure [12, 85]. Patients with symptomatic HFrEF (NYHA II-IV) were excluded in both trials, which means that patients with heart failure at baseline had either asymptomatic HFrEF, HFrEF with NYHA class I, HFmrEF or HFpEF [12, 85]. In the FIDELIO-DKD analysis, 436 (7.7%) of the overall population had a history of heart failure [85]. Over a median-follow-up of 2.6 years, the composite CV outcome of CV-death, non-fatal MI and stroke or hospitalization for heart failure was significantly reduced by finerenone independent of the presence or absence of heart failure (hazard ratio 0.73, 95% CI 0.50–1.06 and hazard ratio 0.90, 95% CI 0.77–1.04, respectively; P = 033) [85]. Similar results were reported in the prespecified analysis of FIGARO-DKD, where 571 (7.8%) patients of the overall population had a history of heart failure at baseline [12]. Consistently, the beneficial effects of finerenone on improving heart failure outcomes were not modified by the presence or absence of heart failure [12]. Finerenone also significantly reduced the incidence of new-onset heart failure when compared to placebo [12]. These data show for the first-time favorable actions of the non-steroidal MRA finerenone in a larger population of heart failure patients with CKD and T2DM. Also, regarding the applicability of these data to patients with HFpEF, these results seem very important. The exclusion of patients with HFrEF in FIDELIO-DKD and FIGARO-DKD increases the likelihood that many of the patients diagnosed in these trials at baseline with heart failure had HFpEF. This is further supported by the baseline characteristics of these patients including more female patients, higher body mass index, and higher C-reactive protein – all signs indicative of HFpEF [12, 85].

Taken together, both the preclinical data and the currently available clinical data with finerenone demonstrate a yet to be precisely defined therapeutic potential of this non-steroidal MRA in patients with HFpEF. Finally, this question will be answered in the ongoing FINEARTS-HF Study to Evaluate the Efficacy and Safety of Finerenone on Morbidity & Mortality in Participants with Heart Failure and LV-Ejection Fraction ≥ 40%) (NCT04435626) (Table 2). Participants with NYHA class II-IV under diuretic treatment, with a documented LV-ejection fraction ≥ 40%, structural heart abnormalities defined by at least one of the following findings: left atrial diameter ≥ 3.8 cm, left atrial area ≥ 20cm^2^, left atrial volume ≥ 30mL/m^2^, left ventricular mass index ≥ 95 g/m^2^ (female)/ 115 mg/m^2^ (male), septal thickness or posterior wall thickness ≥ 1.1 cm and increased blood levels of NT-prBNP will be included [86]. They will be randomized to 20 mg finerenone (eGFR ≤ 60mL/min/1.73m^2^), 40 mg finerenone (eGFR > 60mL/min/1.73m^2^) or placebo [86]. The primary outcome is a composite of cardiovascular death and heart failure events [86]. The study is active, not recruiting anymore and has enrolled 6.016 participants [86]. The estimated study completion date is in August 2024.

## Conclusions

Finerenone is a novel, highly selective non-steroidal MRA with high binding affinity and short plasma half-life. In two major endpoint-driven clinical trials in patients with CKD and T2DM (FIDELIO-DKD and FIGARO-DKD), finerenone induces significant cardiorenal protective actions. Beyond these results, finerenone has shown to improve multiple pathophysiological parameters of HFpEF in preclinical models. In consonance, pre-specified subgroup analyses of FIDELIO-DKD and FIGARO-DKD suggested a potential beneficial effect of finerenone in HFpEF.

HFpEF is a devastating clinical syndrome with increasing prevalence and poor prognosis. Unfortunately, current guideline-recommend drug therapy in HFpEF is very limited with only one prognosis-improving drug class, SGLT2-inhibitors. Against this background, the potential beneficial effects of finerenone in HFpEF appear very promising. The final efficacy/ risk profile of finerenone in HFpEF is currently being investigated in over 6.000 patients in the FINEARTS-HF study, the results of which are desperately awaited to allow this drug to be used in the treatment of HFpEF.

## Data Availability

All data and material will be provided upon request.

## Abbreviations

MR: mineralocorticoid receptor
MRA: MR antagonist
HFrEF: heart failure with reduced ejection fraction
ACE-I: angiotensin converting enzyme inhibitor
ARNI: angiotensin receptor-neprilysin inhibitor
SGLT2: sodium-glucose co-transporter 2
HFpEF: heart failure with preserved ejection fraction
FIDELIO-DKD: The Finerenone in Reducing Kidney Failure and Disease Progression in Diabetic Kidney Disease trial
FIGARO-DKD: The Finerenone in Reducing Cardiovascular Mortality and Morbidity in Diabetic Kidney Disease trials
CKD: chronic kidney disease
T2DM: type 2 diabetes mellitus
CV: cardiovascular
BNP: brain natriuretic peptide
RALES: Randomized Aldactone Evaluation Study
EPHESUS: Eplerenone Post-Acute Myocardial Infarction Heart Failure Efficacy and Survival Study
EMPHASIS: Eplerenone in Mild Patients Hospitalization and Survival Study in Heart Failure
SBP: systolic blood pressure
eGFR: estimated glomerular filtration rate
UACR: urinary albumin to creatinine ratio
CI: confidence interval
NP: natriuretic peptide
NO: nitric oxide
EMPEROR-Preserved: Empaglifozin Outcome Trial in Patients with Chronic Heart Failure with Preserved Ejection Fraction
DELIVER: Dapaglifozin Evaluation to Improve Lives of Patients with Preserved Ejection Fraction Heart Failure
TOPCAT: Treatment of Preserved Cardiac Function Heart Failure with an Aldosterone Antagonist
Aldo-DHF: Aldosterone Receptor Blockade in Diastolic Heart Failure trial
HOMMAGE: Heart Omics in AGEing trial
SPIRRIT-HF: Spironolactone Initiation Registry Randomized Interventional Trial in Heart Failure with Preserved Ejection Fraction
SPIRIT-HF: Spironolactone in the Treatment of Heart Failure trial
HFA: Heart Failure Association
DOCA: deoxycorticosterone acetate
LV: left ventricle
GLS: global longitudinal strain
ARTS: Mineralocorticoid Receptor Antagonist Tolerability Study
ARTS-HF: Mineralocorticoid Receptor Antagonist Tolerability Study–Heart Failure
FINEARTS-HF: Study to Evaluate the Efficacy and Safety of Finerenone on Morbidity & Mortality in Participants with Heart Failure and LV-Ejection Fraction ≥ 40%
